# Comparison of HIV-1 viral load based on RNA or reverse transcriptase activity in patients with suspected viral load underestimation

**DOI:** 10.1186/1742-4690-9-S1-P29

**Published:** 2012-05-25

**Authors:** Beatrice N Vetter, Cyril Shah, Jürg Böni, Jörg Schüpbach

**Affiliations:** 1 Swiss National Center for Retroviruses, Institute for Medical Virology, University of Zürich, Switzerland

## Introduction

In HIV diagnostics, viral load (VL) measurement is based on viral RNA. Occasionally, untreated patients present with a low VL of ≤1000 copies/ml, in which case the VL may be underestimated. In Switzerland, patients with suspected underestimation of VL by nucleic acid testing (NAT) are offered VL assessment by the Product Enhanced Reverse Transcriptase (PERT) assay. Here, we compared VL measurement by these two methods in order to assess the frequency and magnitude of NAT-based VL underestimation.

## Material and methods

We compared VL by PERT and NAT for three different patient groups: (1) newly diagnosed patients with suspected underestimation of VL by NAT (n=19); (2) patients receiving VL monitoring by PERT based on previously confirmed underestimation (n=28), and (3) a reference group of untreated, subtype B-infected patients (n=16). The output of both assays was copies/ml viral RNA. For the PERT assay, conversion to copies/ml was based on a reference correlation of NAT VL and RT-activity (qPCR).

## Results

In newly diagnosed patients approximately 4% have a suspected VL underestimation by NAT (≤1000 copies/ml). PERT results were available for 19 of 59 of such newly diagnosed patients (32.2%). The median difference (log copies/ml) between PERT and NAT VL for this group was 1.36, compared to 0.92 for PERT-monitored patients and -0.004 for the reference group. In 74% of newly diagnosed and 68% of PERT-monitored patients the VL by PERT was ≥5x higher compared to NAT (reference group: 0%). Correlation between PERT and NAT was at R2=0.02 for newly diagnosed patients, 0.63 for PERT-monitored patients and 0.89 for the reference group. Patient groups (1) and (2) both comprised a mixture of subtypes, including subtype B.

## Conclusions

This analysis confirms that VL underestimation still occasionally occurs, even with the improved contemporary VL tests. Causes include sequence variations leading to impaired primer/probe-binding during cDNA amplification. As inadvertent VL underestimation may lead to further infections or inappropriate treatment decisions, a sequence-independent test, like the PERT, remains valuable for confirming a low VL.

